# The synthetic dye malachite green found in food induces cytotoxicity and genotoxicity in four different mammalian cell lines from distinct tissuesw

**DOI:** 10.1093/toxres/tfad059

**Published:** 2023-07-24

**Authors:** Andryo O de Almada Vilhena, Karina M M Lima, Luana F C de Azevedo, Jorge D Rissino, Augusto C P de Souza, Cleusa Y Nagamachi, Julio C Pieczarka

**Affiliations:** Centro de Estudos Avançados da Biodiversidade, Laboratório de Cultura de Células, Instituto de Ciências Biológicas, Universidade Federal do Pará/Parque de Ciência e Tecnologia do Guamá, Avenida Perimetral da Ciência Km 01 – Guamá, Belém CEP 66075-750, PA, Brazil; Instituto Tocantinense Presidente Antônio Carlos (ITPAC/Afya), Abaetetuba, PA, Brazil; Centro de Estudos Avançados da Biodiversidade, Laboratório de Cultura de Células, Instituto de Ciências Biológicas, Universidade Federal do Pará/Parque de Ciência e Tecnologia do Guamá, Avenida Perimetral da Ciência Km 01 – Guamá, Belém CEP 66075-750, PA, Brazil; Campus Tomé Açu, Universidade Federal Rural da Amazônia, Tomé Açu, PA, Brazil; Centro de Estudos Avançados da Biodiversidade, Laboratório de Cultura de Células, Instituto de Ciências Biológicas, Universidade Federal do Pará/Parque de Ciência e Tecnologia do Guamá, Avenida Perimetral da Ciência Km 01 – Guamá, Belém CEP 66075-750, PA, Brazil; Centro de Estudos Avançados da Biodiversidade, Laboratório de Cultura de Células, Instituto de Ciências Biológicas, Universidade Federal do Pará/Parque de Ciência e Tecnologia do Guamá, Avenida Perimetral da Ciência Km 01 – Guamá, Belém CEP 66075-750, PA, Brazil; Centro de Estudos Avançados da Biodiversidade, Laboratório de Cultura de Células, Instituto de Ciências Biológicas, Universidade Federal do Pará/Parque de Ciência e Tecnologia do Guamá, Avenida Perimetral da Ciência Km 01 – Guamá, Belém CEP 66075-750, PA, Brazil; Laboratório de Estudos da Ictiofauna da Amazônia, Campus Abaetetuba, Instituto Federal do Pará, Abaetetuba, PA, Brazil; Centro de Estudos Avançados da Biodiversidade, Laboratório de Cultura de Células, Instituto de Ciências Biológicas, Universidade Federal do Pará/Parque de Ciência e Tecnologia do Guamá, Avenida Perimetral da Ciência Km 01 – Guamá, Belém CEP 66075-750, PA, Brazil; Centro de Estudos Avançados da Biodiversidade, Laboratório de Cultura de Células, Instituto de Ciências Biológicas, Universidade Federal do Pará/Parque de Ciência e Tecnologia do Guamá, Avenida Perimetral da Ciência Km 01 – Guamá, Belém CEP 66075-750, PA, Brazil

**Keywords:** apoptosis/necrosis, micronucleus, MTT, comet assay

## Abstract

Malachite green (MG) is a synthetic dye that uses ranges from its application as a tissue dye to that as an antiparasitic in aquaculture. Several studies have reported the presence of this compound in food dyes and in the meat of fish raised in captivity for human consumption, suggesting risks both for the end user and for as those who handle these products because of MG toxic properties described in the literature. Here we evaluated the cytotoxic and genotoxic profiles of MG in four different cell lines (ACP02, L929, MNP01, and MRC-5). Two of these cell lines are stomach cells (normal and cancer lineages) and the potential ingestion of MG makes this a relevant cell type. Cells were treated with MG at concentrations ranging from 0.1 μM to 100 μM, and tested by MTT assay, a differential apoptosis/necrosis assay (EB/OA), the micronucleus test (MN), and the comet assay. MG exhibits dose-dependent cytotoxicity toward all of the tested cell types; higher concentrations of MG cause cell necrosis, while lower concentrations induce apoptosis. MG has a genotoxic profile increasing the rates of micronuclei, nucleoplasmic bridges, nuclear buds, and DNA fragmentation; L929 and MRC-5 showed more sensibility than ACP02 and MNP01.

**Highlights**

- Malachite green (MG) exhibits a dose-dependent toxic pattern in vitro.
- High concentrations of MG cause apoptosis and necrosis in vitro.
- MG has genotoxic potential, inducing DNA breaks/fragmentation.
- MG must be handled with extreme prudence.

## Introduction

Humans have used pigments since the prehistoric period. The industrial revolution ushered in an increasing demand for dyes, favoring the synthesis of compounds with high yield and coloring power.^1^ Importantly, studies have shown that most of these synthetic dyes present some degree of toxicity to living organisms.^2^^,^^3^^,^^4^^,^^5^

Among the synthetic dyes, the triarylmethane family member, malachite green (MG; C_23_H_25_ClN_2_), is used worldwide as a dye for papers, tissues, and acrylics. Due to its oxidative property, MG has also been used as a disinfectant for surgical materials and as a prophylactic agent for infections in surgical incisions.^6^^,^^7^^,^^8^

Another important use of MG, first reported in the 1930s, is for the aquaculture treatment of external infections on fishes and crustaceans caused by bacteria, protozoa, and/or fungi.^7^^,^^9^^,^^10^^,^^11^ This compound is a potent bioaccumulator^7^^,^^12^ and in vitro studies have shown that high concentrations of MG decrease cell viability, cause DNA damage, and increase the expression of genes linked to cell death.^13^^,^^14^^,^^15^^,^^16^ Thus, the use of MG in treating ichthyo-parasitic infections on aquatic organisms destined for human consumption is strongly discouraged. Indeed, given its well-supported toxic profile, the use of MG in aquaculture is prohibited in most countries around the world. However, MG has been detected in food coloring and fish destined for human consumption.^17^^,^^18^^,^^19^ Moreover, ornamental fish may still be treated with MG, and such users often apply unsuitable methods when handling and discarding it. MG is easily dispersed in the air and its physical and chemical properties render it likely to contaminate handlers, making this agent a problem for human health.^20^^,^^21^^,^^22^^,^^23^^,^^24^ In this context, studies have explored the toxic mechanisms and safety of MG. But here we investigated the cytotoxic and genotoxic potential of MG in four cell lines originating from different tissues and tissue states (ACP02, L929, MNP01, and MRC-5 cells) with the goal of better understanding how different cell types may respond to the toxicological profile of MG.

## Material and methods

### Cell lines

The cell lines used in this research were ACP02 (gastric adenocarcinoma, *Homo sapiens*), L929 (epithelial fibroblast, *Mus musculus*), MNP01 (normal gastric mucosa, *H. sapiens*), and MRC-5 (lung fibroblast, *H. sapiens*). L929 and MRC-5 cells are widely used in toxicology to assess cytotoxicity and oxidative stress.^25^^,^^26^^,^^27^^,^^28^^,^^29^ MNP01 cells, which originated from human stomach,^30^ were selected because MG has been found in food intended for human consumption. ACP02 cells^31^ were used to enable comparison between normal and cancer cells arising from the human stomach. The cell lines MNP01 and ACP02 were kindly provided by Dr Rommel Burbano (Banco de Células HUJBB/UFPA). The cells were cultured in Dulbecco’s Modified Eagle’s Medium (DMEM, Gibco) supplemented with fetal bovine serum (10%, Gibco), PenStrep (100 IU/mL; Gibco) and amphotericin B (2.5 μg/mL; Cristalia) in an incubator (UltraSafe) with a 5% CO_2_ atmosphere.

### Cytotoxicity assay

Cell viability was assessed using the MTT (3-(4,5-dimethylthiazol-2-yl)-2,5-diphenyl-2H-tetrazolium bromide) described by,^32^ with modifications. A total of ⁓6.7 × 10^3^ cells/well were seeded to 96-well plates and treated with 100, 50, 25, 10, 5, 1, or 0.1 μM of MG for 24, 48, or 72 h. These concentrations were determined according to the gradient proposed by Stammati et al.^15^ An equal volume of DMEM was used as a negative control (NC). At the end of the treatment period, the medium was removed, the cells were exposed to 100 μL of MTT salt (0.5 mg/mL) for 3 h, and then 100 μL of DMSO (Dimethyl sulfoxide) was added for 1 h. Absorbance was recorded using a spectrophotometer (BioTek) at 570 nm. Cell viability was expressed as a percentage (%) and the IC_50_ (inhibitory concentration 50%) was determined using the GraphPad Prism 8 software (GraphPad Software Inc., San Diego, California). Then we determined sublethal concentrations based on IC_50_ values to each strain used ensuing assays (Table 1).

**Table 1:** Sublethal concentrations used in subsequent assays based on IC_50_ values obtained from cytotoxicity MTT assay.

	IC_50_	IC_50(24h)_	IC_50(48h)_
ACP02	24 h: 36.91 μM 48 h: 34.12 μM	C1 (25%): 9.22 μM	C1 (25%): 8.53 μM
		C2 (50%): 18.45 μM	C2 (50%): 17.06 μM
		C3 (75%): 27.68 μM	C3 (75%): 25.59 μM
L929	24 h: 14.59 μM 48 h: 1.96 μM	C1 (25%): 3.64 μM	C1 (25%): 1.47 μM
		C2 (50%): 7.29 μM	C2 (50%): 0.98 μM
		C3 (75%): 10.94 μM	C3 (75%): 1.47 μM
MNP01	24 h: 17.62 μM 48 h: 9.52 μM	C1 (25%): 4.40 μM	C1 (25%): 2.38 μM
		C2 (50%): 8.81 μM	C2 (50%): 4.76 μM
		C3 (75%): 13.21 μM	C3 (75%): 7.14 μM
MRC-5	24 h: 5.86 μM 48 h: 0.90 μM	C1 (25%): 1.46 μM	C1 (25%): 0.22 μM
		C2 (50%): 2.93 μM	C2 (50%): 0.45 μM
		C3 (75%): 4.39 μM	C3 (75%): 0.67 μM

### Differential assay for apoptosis/necrosis

**Fig. 1:**
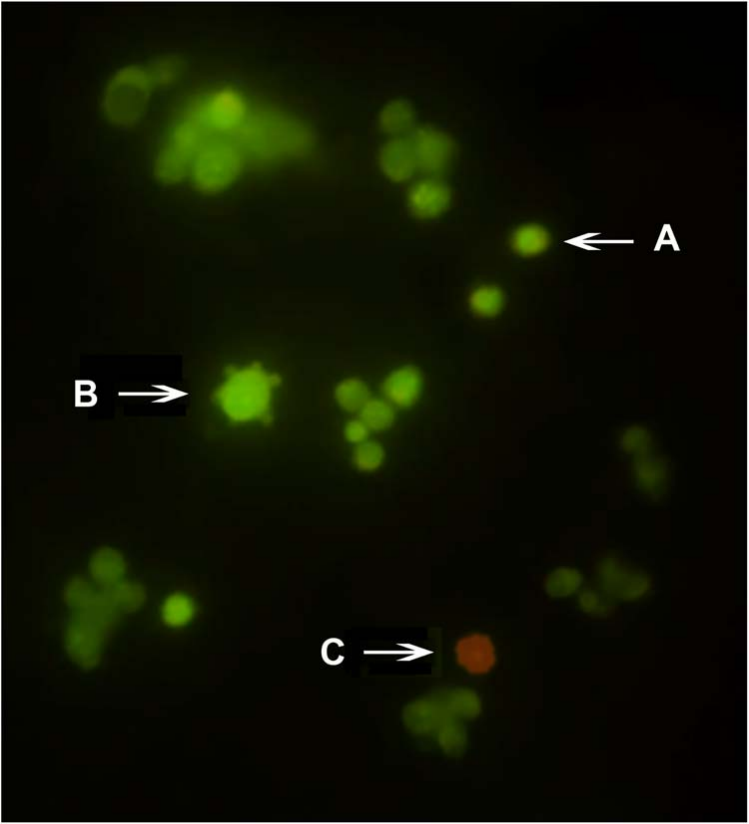
Standard staining with ethidium bromide and acridine orange (EB/AO). (A) Viable cell; (B) Cell in apoptosis; (C) Necrotic cell.

Cells (8 × 10^4^ per well) were seeded in 12-well plates, incubated for 24 h, and exposed to sublethal concentrations corresponding to 25%, 50%, and 75% of the above-determined IC_50(24 h)_. Positive control (PC) wells were treated with doxorubicin (100 μg/mL) and NC wells were treated with DMEM alone. After 24 h of exposure, the cells were trypsinized, centrifuged, and collected, and 20.0 μL of the cell-containing suspension was mixed with 2 μL of a solution containing ethidium bromide (100 μg/mL) and acridine orange (100 μg/mL). The cells were then applied to glass slides and immediately analyzed under a fluorescence microscope (Nikon). We scored 300 cells per concentration in duplicate according to the standards established by Alcântara et al.^33^ and de Azevedo et al.^34^: cells with intact green-stained chromatin were considered viable cells; cells with fragmented green-stained chromatin and apoptotic corpuscles were considered to be apoptotic cells; and cells with red-stained chromatin and no evidence of nuclear condensation were considered to be necrotic cells (Fig. 1).

### Micronucleus assay with cytokinesis block

The micronucleus assay was performed to verify mutagenicity (clastogenicity or aneugenicity), according to the protocol described by Fenech^35^, with adaptations. Cells (~8 × 10^4^ per well) were seeded to 12-well plates, incubated for 24 h in the CO_2_ incubator at 37°C, and exposed to the MG sublethal concentrations determined by MTT assay for each strain. The exposure was made for 48 h. PC cells were treated with colchicine (0.02 μg/mL)^36^ and NC cells were treated with DMEM alone. Cytochalasin B (6 μg/mL) was added 24 h before the end of the experiment. After that, the cells were trypsinized and centrifuged, and the supernatant was discarded. Each cell pellet was mixed with hypotonic solution (1 M; KCl) for 3 min and then 2 mL of Carnoy’s fixative solution was added in order to stop hypotonic activity. The analysis was performed in duplicate as a blind test. A total of 500 cells were quantified to determine the cytokinesis-block proliferation index (CBPI), which was calculated as follows: [(n° of mononuclear cells) + (2 × n° of binucleate cells) + (3 × n° multinucleated cells)/total number of cells. For binucleate cells, 1,000 cells per well were examined for micronuclei and other nuclear abnormalities; frequencies were calculated using the formula: ƒabnormality = (number of abnormalities/1,000).

### Comet assay

The comet assay was performed to check primary DNA damage according to the protocol described by Singh et al.^37^, with modifications. Cells (~8 × 10^4^ per well) were seeded to 12-well plates, incubated for 24 h in the CO_2_ incubator at 37°C, and then exposed for 3 h to three sublethal concentrations of MG, also determined by MTT assay for each strain. PC wells were treated with doxorubicin (5 μg/mL); NC wells were treated with DMEM alone. After that, the cells were then trypsinized and centrifuged, and the supernatant was discarded. Each pellet was resuspended with 80 μL of *low melting point* agarose (0.5%) and this solution was laid on glass slides previously covered with normal agarose (1.5%), then covered with coverslips and incubated for 15 min at 4°C and placed in lysis solution (2.5 M NaCl, 100 mM EDTA and 10 mM Tris [pH 10.0–10.5]) with 1% Triton X-100 and 10% DMSO. After 24 h, the slides were submerged in an alkaline solution (300 mM NaOH and 1 mM EDTA, pH > 13) for 30 min and electrophoresed (26 V, 300 mA, 25 min). The cells were fixed with ethanol and stained with ethidium bromide (10 μg/mL). A total of 100 cells were analyzed in duplicate for each concentration, using a fluorescence microscope (Nikon) with 40× magnification. Comets were classified in four categories, based on the size of the tail.^36^ The damage index (DI) was estimated using the formula: DI = [(1^x^n1) + (2^x^n2) + (3^x^n3) + (4^x^n4)/n] × 100, where *n* represents the total cells analyzed and n1 to n4 indicate the numbers of cells with damage levels of 0–4.

### Statistical analysis

Normality, variance, and linear regression analyses were performed using the BioEstat 5.3 and GraphPad Prism 8 software packages (GraphPad Software Inc., San Diego, California). Normality was defined by the Kolmogorov–Smirnov test and multiple comparisons were performed using ANOVA followed by the Tukey test. A value of *P* < 0.05 was adopted as representing a statistically significant difference.

## Results

### Cell viability

**Fig. 2:**
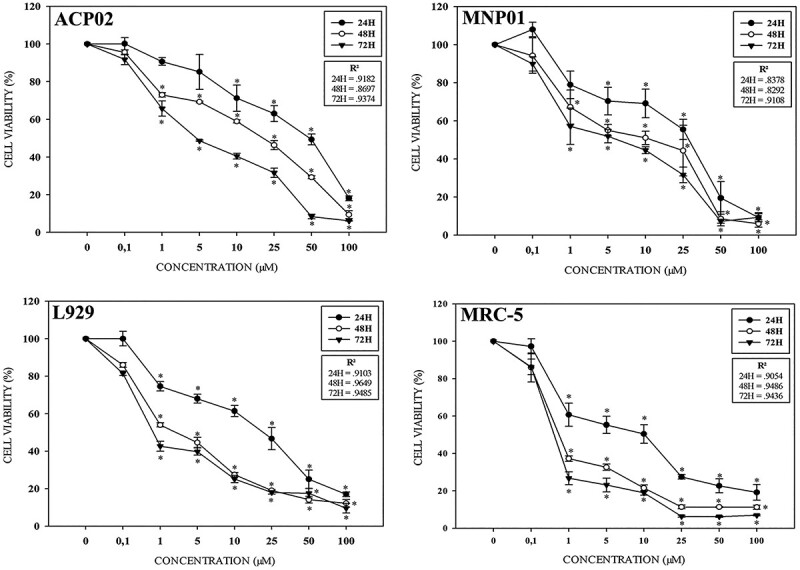
Cell viability (%) of lineages exposed to different concentrations (μM) of MG for 24, 48, and 72 h. The R^2^ values obtained from linear regression analyses support a strong correlation between exposure time, MG concentration, and cell viability rate; these results indicate that MG exhibits dose-dependent toxicity. Asterisks (*) indicate *P* < 0.05 when compared to the control group (ANOVA; Tukey test for multiple comparisons).

The results obtained from MTT assay showed that among the four lineages tested, ACP02 exhibited the highest resistance (Fig. 2A), while MRC-5 demonstrates the lowest resistance. Regarding the tested concentrations, we observed that the lowest concentration (0.1 μM) does not exert cytotoxicity on the cells at any time tested, however, from the concentrations above 1.0 μM it is already possible to visualize the dose-dependent reduction in viability in all strains and exposure times. It is also possible to observe that higher concentrations and exposure times reflect greater reductions in viability (Fig. 2A–D).

**Fig. 3:**
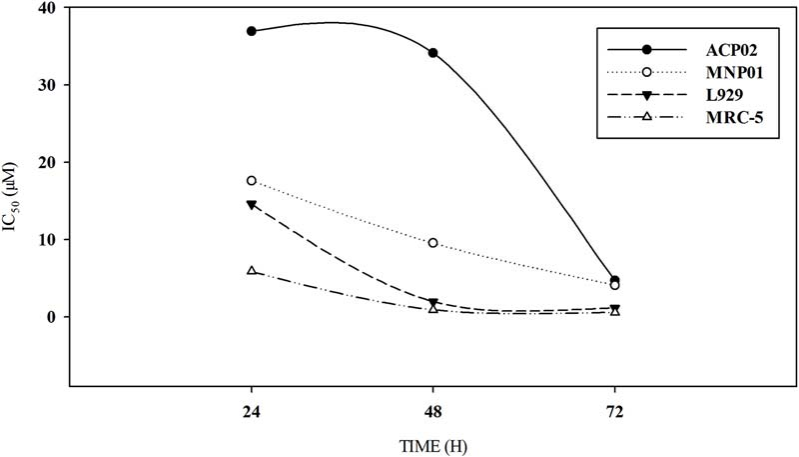
Comparison of different IC_50_ values calculated from MTT assay-based cell viability results, as calculated using the GraphPad prism 8 software.

By comparing the IC_50_ (Fig. 3), it becomes possible to better visualize the different responses in the tested cells. After 24 h of treatment, the IC_50_ values for MG were 36.91, 17.62, 14.59, and 5.86 μM for ACP02, MNP01, L929, and MRC-5 cells, respectively (Fig. 3). After 48 h of treatment, the IC_50_ values were 34.12, 9.52, 1.96, and 0.90 μM, respectively. After 72 h, there was a narrower variation in the IC_50_ values, which were 4.68, 4.08, 1.15, and 0.57 μM, respectively.

### Apoptosis/necrosis: Differential assay with ethidium bromide and acridine orange

**Fig. 4:**
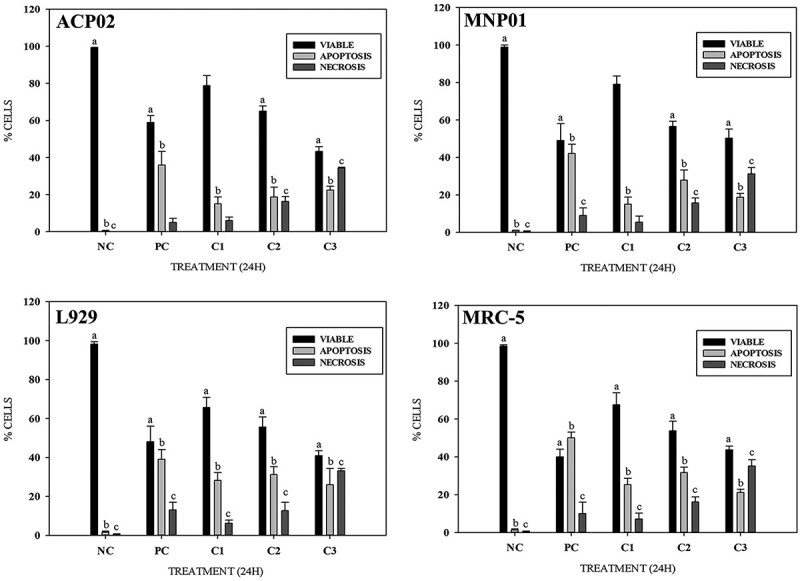
Rate (%) of viable, apoptotic, and necrotic cells following treatment with sublethal concentrations of MG for 24 h stained with ethidium bromide and acridine orange (EB/AO). C1: IC50_(24h)(25%)_; C2: IC_50(24h)(50%)_; and C3: IC_50(24h)(75%)_. Bars with the same letter represent *P* < 0.05 when compared to the control group (ANOVA; Tukey test for multiple comparisons).

The 24-h exposure to the lowest concentration of MG (IC_50(24h)(25%)_) increased the numbers of apoptotic cells in all four cell lines. The intermediate concentration (IC_50(24h)(50%)_) caused both apoptosis and necrosis, with markedly higher numbers of necrotic cells seen in L929 and MRC-5 cells. The highest concentration (IC_50(24h)(75%)_) caused considerable increases in the number of necrotic cells in all tested cell lines (Fig. 4).

### Micronucleus assay with cytokinesis block

When MG was applied at the lowest concentration (IC_50(48h)(25%)_), there was no significant change in the frequencies of MN, nucleoplasmic bridges, or nuclear buds in any of the tested cell lines. Intermediate treatment (IC_50(48h)(50%)_) significantly increased the frequencies of bridges and buds in L929 cells, that of MN in MNP01 cells, and that of bridges in MRC-5 cells. It is observed that highest (IC_50(48h)(75%)_) treatment increases the frequencies of MN, bridges and buds in all cell lines. Significant decreases in CPBI were seen when all of the tested cell lines were treated with the highest concentration of MG (IC_50(48h)(75%)_) (Table 2).

### Comet assay

**Fig. 5:**
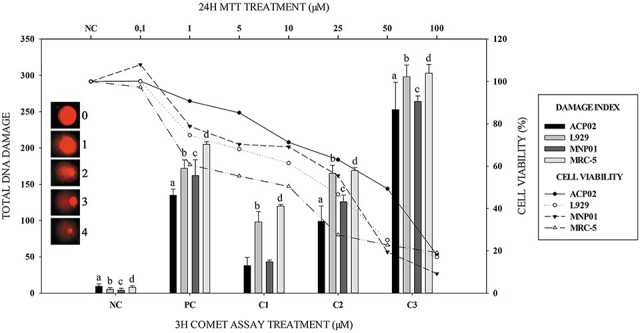
Total DI (bars) characterized by comet assay analysis of cells exposed to sublethal concentrations of MG. C1: IC_50(24h)(25%)_; C2: IC_50(24h)(50%)_; and C3: IC_50(24h)(75%)._ Asterisks (*) represent *P* < 0.05 when compared to the control group (ANOVA; Tukey test for multiple comparisons).

Among cells treated with the lowest concentrations of MG (C1: IC_50(24h)(25%)_), the MRC-5 and L929 cell lines exhibited significant DNA fragmentation (*P* < 0.05). The intermediate and highest concentrations (C2: IC_50(24h)(50%)_ and C3: IC_50(24h)(75%)_) conferred significant damage to all treated cell lines, compared to the NC (*P* < 0.05). Figure 5 shows the DNA fragmentation index and its relationship with cell viability in cells treated with different concentrations of MG for 24 h exposure.

## Discussion

In vitro assays are widely used in toxicology due their speed and accuracy.^38^ Studies have evaluated the cytotoxicity of MG against cells from different organisms, including fish,^39^ rodents,^40^^,^^41^ and humans (normal and cancer cells),^15^ being reported cytotoxicity, oxidative tumorigenic and carcinogenic potential in vivo. However, no previous study has compared the MG sensitivity/resistance of different cell lines obtained from normal and cancerous mammalian tissues. Regarding the tissue of origin, cancerous stomach cells (ACP02) were more resistant than normal stomach cells (MNP01), and the latter were more resistant than subcutaneous epithelium (L929) and pulmonary fibroblasts (MRC-5).

Studies about effects of MG on living organisms are scarce, however research has reported genotoxic effects on fish (*Hemichromis bimaculatus*)^42^; in female mice (B6C3F1 lineage) traces of lymphocytic genotoxicity were not reported, however this compound causes transplantation in liver cells, characterizing a carcinogenic potential, probably due its metabolization.^43^

The results observed in the ACP02 strain when exposed to MG were already expected, as it is a diffuse cell type that is proven to exhibit high resistance to exogenous components.^44^^,^^45^ Cancer cells generally exhibit greater resistance to cytotoxic compounds; this arises from cancer-related modifications in the genome that lead to resistance against cell death.^46^^,^^47^ In particular, ACP02 cells harbor deletions of the tumor suppressor genes, TP53 and FBXW7 specifically, which are the main factors related to the ability to escape from apoptosis.^48^ Deregulation of cell death processes disrupts tissue and/or organ homeostasis, leading to several conditions, including carcinogenesis.^47^^,^^48^^,^^49^^,^^50^ An analogous study^15^ used the MTT test to determine the IC_50(24h)_ of Caco-2 cells (a colorectal adenocarcinoma cell line) treated with 16.2 μM of MG. In the present work, we found that the IC_50(24h)_ of ACP02 cells was twice that seen in Caco-2 cells, and that these levels of cytotoxicity were higher than those obtained in cells derived from normal tissues (MNP01, L929, and MRC-5).

We propose that slight toxicity observed between the L929 and MRC-5 strains is due to molecular and biochemical characteristics inherent to each cell type, allowing them to have different levels of tolerability to MG, a phenomenon known as selective toxicity.^53^

Low concentrations of MG increased the differential expression of genes linked to apoptosis and oxidative stress in cultured cells,^16^ and that this is likely to be related to lipid peroxidation.^54^^,^^55^^,^^56^ This process leads to the formation of reactive oxygen species (ROS), which harm DNA when present at high levels, resulting in activation of metabolic pathways leading to apoptosis.^51^^,^^52^ Furthermore, ROS have oxidative activity and, at high levels, can facilitate necrosis by destabilizing and disrupting the plasma membrane^54^ corroborating the results observed apoptosis/necrosis and comet assay in present study. 

**Table 2:** Frequency of micronuclei (MN), nucleoplasmic bridges, and nuclear buds in cells treated with sublethal concentrations of MG for 48 h. C1: IC_50(48h)(25%)_; C2: IC_50(48h)(50%)_; and C3: IC_50(48h)(75%)._ Asterisks (*) indicate *P* < 0.05 when compared to the control group (ANOVA; Tukey test for multiple comparisons).

	Treatment	CBPI	ƒMNs	ƒBridges	ƒBuds
ACP02	*NC*	1.532(±0.081)	0.004(±0.003)	0.000(±0.000)	0.000(±0.000)
	*PC*	1.516(±0.061)	0.034(±0.011)*	0.000(±0.000)	0.003(±0.001)
	*C1*	1.495(±0.076)	0.016(±0.006)	0.005(±0.004)	0.005(±0.007)
	*C2*	1.472(±0.076)	0.015(±0.006)	0.005(±0.003)	0.010(±0.003)
	*C3*	1.237(±0.052)*	0.029(±0.014)*	0.017(±0.006)*	0.016(±0.006)*
L929	*NC*	1.425(±0.040)	0.001(±0.001)	0.001(±0.000)	0.001(±0.000)
	*PC*	1.380(±0.091)	0.019(±0.005)*	0.001(±0.001)	0.001(±0.001)
	*C1*	1.384(±0.045)	0.004(±0.002)	0.013(±0.002)	0.009(±0.002)
	*C2*	1.326(±0.052)	0.005(±0.003)	0.008(±0.004)*	0.013(±0.005)*
	*C3*	1.173(±0.051)*	0.013(±0.005)*	0.013(±0.004)*	0.018(±0.006)*
MNP01	*NC*	1.467(±0.057)	0.001(±0.001)	0.000(±0.000)	0.000(±0.000)
	*PC*	1.477(±0.065)	0.038(±0.006)*	0.002(±0.001)	0.002(±0.001)
	*C1*	1.480(±0.012)	0.006(±0.003)	0.007(±0.005)	0.006(±0.004)
	*C2*	1.344(±0.042)*	0.014(±0.006)*	0.010(±0.006)	0.007(±0.003)
	*C3*	1.212(±0.041)*	0.020(±0.007)*	0.016(±0.008)*	0.010(±0.005)*
MRC-5	*NC*	1.423(±0.027)	0.001(±0.001)	0.001(±0.000)	0.001(±0.000)
	*PC*	1.404(±0.097)	0.019(±0.005)*	0.001(±0.001)	0.001(±0.001)
	*C1*	1.322(±0.069)	0.004(±0.002)	0.013(±0.002)	0.009(±0.002)
	*C2*	1.214(±0.046)*	0.005(±0.003)	0.008(±0.004)*	0.013(±0.005)
	*C3*	1.156(±0.046)*	0.013(±0.005)*	0.013(±0.004)*	0.018(±0.006)*

The result observed in MN test corroborates previous reports indicating that high concentrations of MG increase the frequency of MN and other chromosomal abnormalities.^55^^,^^57^ Thus, MG is considered to be a potential genotoxic agent that can generate chromosomal changes, which can lead to tumor formation and possibly cancer.^58^^,^^59^ Two other markers for genotoxicity are significant increases in nuclear buds, which are structures reportedly linked to the process of gene extrusion for the elimination of badly repaired DNA,^60^^,^^61^ and nucleoplasmic bridges, which arise from chromosome fragility and are associated with chromosomal rearrangements.^62^^,^^63^ The results of these other two markers reinforce the possibility of carcinogenic potential reported by other authors.^7^^,^^21^^,^^64^^,^^65^

The data from the MN test and the comet assay can be correlated,^66^ but the latter further reveals damage that may still be subject to correction by the DNA repair machinery.^67^ In the present study, L929 and MRC-5 cells treated with the lowest concentration of MG showed differences between the MN test and the comet assay. We speculate that the DNA fragmentations observed in the comet assay results from these two cell lines have been repaired after a cell division cycle, such that there is no evidence of MN formation or another nuclear abnormality thereafter. Cells exposed to MG tend to stagnate in G2/M phase, suggesting that they undergo a temporal extension of checkpoint repair.^68^ This could also be related to observed decrease in CPBI, as assessed by the MN test with highest MG concentrations.

The results of our alkaline comet assay, which evaluates single- and double-strand breaks before the action of repair systems, are consistent with the idea that MG has physical and chemical properties that allow it to interact with and fragment DNA—this was previously proposed by Bhasikuttan et al.^69^ who used photochemical methods to demonstrate an interaction between MG and DNA. The authors suggested that MG interacts intensively with DNA in certain GC-rich segments of the nucleotide sequence, particularly oligomeric regions with the sequence d(G_2_T)_13_G (called the “G-quadruplex”). Cationic compounds like MG tend to form covalent bonds with anionic sites,^70^ such as the phosphate ions of the DNA structure. Considering the possible interactions between MG and DNA coupled with the dose-dependent toxicological pattern of MG, we hypothesize that excess MG-DNA interactions cause genomic instability, as evidenced by increase chromosomal breakage and DNA fragmentation. In a similar work, Fessard et al.^14^ studied CHO cells (Chinese hamster ovary) and observed that high concentrations of MG decreased cell viability and increased the rate of DNA breaks, and thus obtained results similar to those of the present study. In this context it is relevant that^71^ showed that cells with severe genomic damage trend toward apoptosis, causing distress to tissue/organ homeostasis. It is worth emphasizing that in the present work was developed with different cell lines from distinct tissues, including stomach cells, thereby potential ingestion, contact or inhalation of MG are dangerous.

## Conclusion

We add to research knowledge by exploring the cell- and DNA-level interactions of MG in four cell lines representing different tissues or tissue states. Under the utilized conditions, we found that MG concentrations below 1.0 μM had no cytotoxic effect on the tested cell lines and that, at its cytotoxicity-inducing concentrations, MG caused apoptosis at the lower concentration range and necrosis at the higher concentration range. Regarding the genotoxicity of MG at sublethal concentrations, this compound showed a high genotoxic profile at high concentrations in all four tested cell lines, with L929 and MRC-5 cells also exhibiting sensitivity at lower concentrations. Specialized cells have different molecular features in terms of proteins in plasma membrane and cytoplasmic enzymes, which can confer different degrees of sensitivity to xenobionts. Our results demonstrated the damage cause by MG in stomach, pulmonary and epithelial cells, both cancerous and non-cancerous, which allows us to conclude that MG confers risks to human health, and that it should always be handled while using personal protective equipment. By the same reason the use of MG in aquatic organisms destined for human consumption and its presence in industrialized foods should be banned (and those bans should be enforced), and disposal should be carried out properly.

## Data Availability

All results reported in this article are found in the main text.
